# Efficacy of an aloe vera, chamomile, and thyme cosmetic cream for the prophylaxis and treatment of mild dermatitis induced by radiation therapy in breast cancer patients: a controlled clinical trial (Alantel Trials)

**DOI:** 10.1186/s13063-024-07901-8

**Published:** 2024-01-25

**Authors:** Celia Jimenez-Garcia, Luis Angel Perula-de Torres, Enrique Villegas-Becerril, Juan Jose Muñoz-Gavilan, Maria Espinosa-Calvo, Gertrudis Montes-Redondo, Esperanza Romero-Rodriguez, Alantel Trial Collaborative Group, Maria Carmen Moreno Manzanaro, Fatima Ginés Santiago, Carmen Bueno Serrano, Fabiola Romero Ruperto, Maria Cruz Linares Ramirez, Maria Angeles Quesada Román, Nieves Muñoz Alcaraz, Juan Manuel Parras Rejano, Maria Isabel Lopez Estepa, Maria Dolores Maestre-Serrano, Jaime Monserrat Villatoro

**Affiliations:** 1 Epidemiology Service, Cordoba-Guadalquivir Health District, Cordoba, Spain; 2 Maimonides Institute for Biomedical Research (IMIBIC)/Hospital Reina Sofia/Universidad de Cordoba, Cordoba, Spain; 3 Maser Clinic, Maimonides Institute for Biomedical Research (IMIBIC), Cordoba, Spain; 4 Lucano Health Center, Cordoba-Guadalquivir Health District, Cordoba, Spain; 5 Radiation Oncology Service, Reina Sofia University Hospital, Cordoba, Spain; 6 Santa Rosa Health Center, Cordoba-Guadalquivir Health District, Cordoba, Spain; 7 Carlos Castilla del Pino Health Center, Cordoba-Guadalquivir Health District, Cordoba, Spain

**Keywords:** Chamomile, Aloe vera, Thyme, Prophylaxis, Dermatitis, Radiation therapy, Oncology, Breast cancer

## Abstract

**Background:**

Dermatitis is a skin condition caused by multiple causes, including radiotherapy treatment. Pharmacological treatments can become chronic and are not exempt from side effects. The latest recommendations of the American Academy of Dermatology establish the use of natural, nourishing, and moisturizing cosmetic products as prevention and the first therapeutic step for dermatitis. Alantel® is a cream developed to reduce redness and irritation, promote the local immune system, combat immunosenescence, and promote the healing of epidermal lesions. The objective was to evaluate the effect of a cream (Alantel) based on natural products at high concentrations for the preventive and curative treatment (at early stages) of radiation-induced dermatitis in patients with breast cancer.

**Methods:**

Our protocol is an experimental, prospective, triple-blind, multicenter, controlled clinical trial with two parallel arms. The experimental group will be treated with Alantel, while the control group will receive another moisturizing cream. Radiotherapy oncology professionals will recruit a total of 88 patients (44 per comparison group) with breast cancer who will receive radiotherapy oncology treatment for 15 days, and they will be randomly allocated to the experimental or control group. Selected patients will be followed up for four visits by primary care physicians for up to 1 week after completion of radiotherapy. The main study variable will be the incidence rate of mild post-radiation dermatitis. An intention-to-treat analysis will be performed, applying a comparison test for independent means and proportions. A bivariate and multivariate analysis will also be developed to check the treatment effect, adjusting for predictive sociodemographic and clinical variables.

**Discussion:**

By carrying out this clinical trial, it is expected to verify that Alantel cream, based on natural products at high concentrations, has advantages over a moisturizing cream for the preventive and curative treatment of RD in patients with breast cancer. The COVID-19 pandemic has been influenced by delaying the start of the study. One of the main limitations of this study will be the time required to recruit the patients from the planned sample, given that the selection criteria are restrictive and, although the study is multicenter, recruitment will be coordinated through a single service on radiotherapy oncology.

**Trial registration:**

ClinicalTrials.gov NCT04116151. Registered on 4 October 2019.

## Introduction

### Background and rationale

Radiotherapy, the use of high-energy rays to either kill cancer cells or treat some benign tumors, is undoubtedly a positive intervention. However, as the primary mode of action in radiotherapy treatment is killing cells to prevent replication, other non-cancerous cells may be affected. For example, up to 85% of patients will experience some form of skin reaction, ranging from local erythema to moist desquamation. Such reactions are distressing and painful for the patient, and if severe enough, they may warrant treatment discontinuation [1].

According to the MSD manual [1], general treatment of dermatitis includes supportive care of cosmetic type (such as moisturizers, dressings, and antihistamines for itching), topical corticosteroids, and sometimes antibiotics or other drugs, or ultraviolet light therapy. Treatment of dermatitis depends on the cause and the specific symptoms. Depending on the severity of the process, this therapeutic ladder can be gradually climbed to treat the milder symptoms to the more severe and chronic ones.

According to the Society and College of Radiographers [2], topical emollients are commonly used to prevent radiation-induced dermatitis (RD) or to provide comfort for patients once a reaction has occurred. As radiation damages the basal cell layer of the skin, the normal desquamation of cells and growth of replacement cells are interrupted, and skin dehydration occurs. Topical emollients are used to hydrate the skin and ameliorate feelings of itching and soreness.

From this point on, all treatments are based on drugs and not cosmetics. However, the latter will serve on the following therapeutic steps to supplement hydration and soothe irritation and pruritus. Among these natural products are aloe vera and chamomile [3–5]. These agents, qualified as cosmetics, have been used to treat iatrogenic RD to alleviate the symptoms and signs of this condition [6–9]. Other studies report using other cosmetic therapies to relieve symptoms and signs of dermatitis [10, 11].

Cosmetics are the first level of treatment required for dermatitis [2]. In the case of RD, several studies report the prevention or treatment of RD with the application of skin products, including aloe vera gel [12], phospholipid-based anionic cream [13], wound healing ointment [14], corticosteroid therapy [15], or hyaluronic acid-based formulation [16]. In addition, washing the skin with soap and water during radiation therapy for cancer treatment may be helpful because it may eliminate various microorganisms that cause skin inflammation [17]. To date, however, no standard therapy or broad consensus on the optimal management of RD in cancer patients is available.

The Radiation Therapy Oncology Working Group has developed a scale of skin lesions after radiation therapy [18]. It comprises 5 degrees scored in ascending order based on severity. Acute or moderate injuries, which would be treatable with cosmetics, include categories 1 and 2: mild atrophy, mild hair loss, mild depigmentation, patch atrophy, and moderate telangiectasia.

However, we have found few clinical trials supporting or demonstrating the efficacy of these cosmetic products for treating RD. Therefore, we believe that providing scientific evidence about this is very appropriate and convenient.

Following these clinical protocols [1, 2] and the literature review on the effectiveness of cosmetics for hydration and relief of symptoms and signs of RD, we consider it relevant to investigate if a cream containing natural products is effective as a prophylactic procedure in breast cancer patients treated with radiation therapy. We focused on this type of cancer as it is one of the most frequent, and in which more patients could benefit if demonstrated that this product is useful. This could avoid climbing the therapeutic ladder toward treating RD with drugs that may lead to side effects. The current drugs cause a pharmaceutical expense that is mostly unproductive because these alone do not adequately manage nutrition, hydration, or de-epithelialization because they do not have the correct formulation. In addition, these treatments are not exempt from side effects, leading to symptoms continuing in the short to medium term.

## Objectives

A controlled clinical trial is planned to objectively evaluate the efficacy of a cosmetic cream. The purpose is to demonstrate that a topical preparation based on active ingredients of natural origin (Alantel®, based on aloe vera, chamomile, and thyme), with anti-inflammatory and epithelization properties, is effective (compared to a harmless product made up of a moisturizing substance), in prophylaxis or curative treatment of mild RD in radiation oncology used to treat breast cancer patients. We also want to assess the safety of this preparation.

As secondary objectives, we consider the following: (a) to check whether this preparation can reduce the discomfort caused by inflammation and cell death, which are the cause of the symptoms and morbidity of radiation-induced dermal lesions in radiation oncology; (b) to determine the health-related quality of life before and after treatment in women who are prescribed this cosmetic cream compared with women who do not use this cosmetic cream; (c) to demonstrate that the coordinated follow-up of this type of patients between Radiation Oncology and Primary Care improves its quality and the health care of these patients.

## Trial design

The Alantel study is designed as a randomized, controlled, multicenter, superiority, triple-blind trial, with two parallel groups.

### Methods: participants, interventions, and outcomes

#### Study setting

**Fig. 1:**
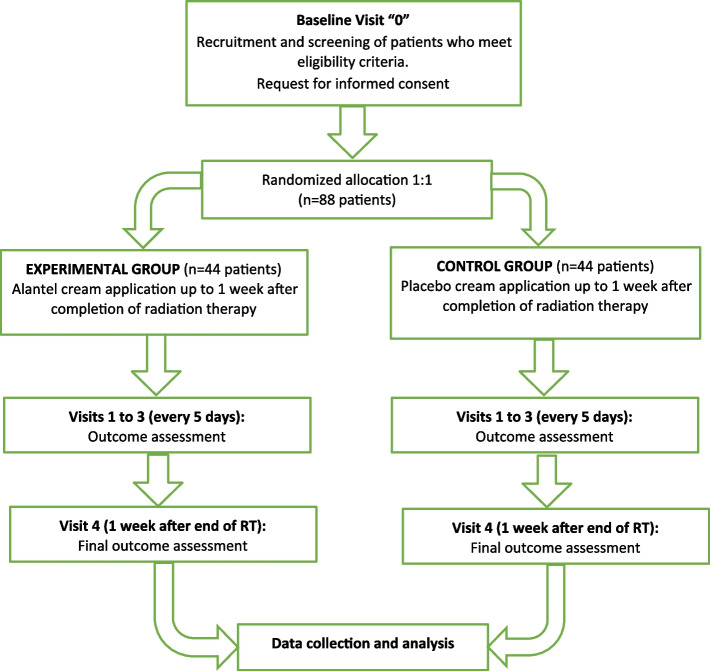
Flowchart of the research phases of the Alantel study

A total of 88 breast cancer patients undergoing radiation therapy will be recruited into the radiation oncology clinic and allocated to the experimental group (EG: Alantel) or the control group (CG: moisturizing cream), with an allocation ratio of 1:1 (see flowchart; Fig. 1). Both groups will receive a 15-session (3 weeks) hypofractionated radiation therapy, subject to the usual hygienic recommendations.

Table 1 shows the schedule of the main events of the study, both the recruitment process and the intervention, and follow-up and evaluation visits.

**Table 1:** Schedule of enrolment, interventions, and assessments from the Alantel study

Time point	Enrolment	Allocation	Study period			Close-out
			Post-Allocation			
	*t* = 0	*t* = 0	*t*1 = 5 days Start RT	*t*2 = 10 days Start RT	*t*3 = 15 days Start RT	*t*4 = 22 days After RT
**Enrolment:**						
Eligibility screen	x					
Informed consent	x					
Allocation		x				
**Interventions:**						
Alantel (Experimental group)		x	x	x	x	x
Placebo (Control group)		x	x	x	x	x
**Assessments**						
**List baseline variables**		x				
Assigned group		x				
Sociodemographic data		x				
Previous history of dermatitis		x				
**List outcome variables**						
Presence/absence of radiodermatitis (RD)			x	x	x	x
Toxicity Level (ORTG Criteria)			x	x	x	x
Degree of discomfort with RD			x	x	x	x
Quality of life related to health			x			x
Presence of adverse events			x	x	x	x
**List other data variables**						
Symptoms and signs of RD			x	x	x	x
RD evolution time			x	x	x	x

The study is conducted at the Radiotherapy Oncology Center of the Reina Sofia Hospital and five participating Primary Care Centers. They are located in Cordoba (Spain) and depend on the Andalusian Health Service.

#### Eligibility criteria

- Inclusion criteria are as follows: patients aged 18 years or older diagnosed with breast cancer and who are going to start radiation therapy with a radical intention on the affected breast, by the hypofractionated scheme with integrated boost (40.05 Gy with integrated boost, 48 Gy, in 15 sessions), coming from centers involved in the project or that can be followed up by a participating primary care professional and that sign an informed consent.
- Exclusion criteria are as follows: patients with dermal lesions or invasive skin cancer or distant metastases; history of connective tissue disorders; severe mental disorder (dementia, drug addiction, etc.); history of hypersensitivity reaction to any of the ingredients in the study cream; history of severe/extensive burn, moisture, erosion, or drainage in the treatment area; or participants involved in other clinical trials within that month.

Fourteen collaborating clinical researchers (5 oncologists, 8 GP, and one primary care occupational therapist) will perform the therapeutic intervention to be tested.

#### Who will take informed consent?

The radiation oncologists will collect the informed consent during the recruitment process. In the informed consent form, in case the recruited patients consent to participate, they will be asked if they accept the use of their data, even if they decide to drop out.

#### Interventions

Once the patient has been included in the study and allocated to one of the two groups, the radiation oncologists will collect demographics and existing pathologies and perform an initial assessment of the skin to be irradiated before the start of radiation therapy. The radiation oncologist will administer the patient a Quality-of-Life Assessment Questionnaire (Skindex-29) [19] and inform the family physician of the patient’s details, identification code, and probable date of initiation of radiation therapy so that the family physician can contact the patient prior to the start of the treatment to schedule follow-up visits.

At each follow-up visit after sessions 5, 10, and 15 and 1 week after completion of radiation therapy, the family physician will perform the following actions:

- Evaluate patients’ irradiated skin according to the common toxicity criteria of the cooperative skin group suggested by the RTOG Foundation (https://www.rtog.org) [18].
- Likewise, patients presenting with dermatitis will be asked to assess and quantify the discomfort and pain related to dermatitis symptoms and signs on an analog ordinal scale (from 1 to 10).
- Complete the Skindex-29 Quality of Life Questionnaire [19] (https://www.bibliopro.org/buscador/294/cuestionario-dermatologico-de-calidad-de-vida-skindex-29).
- Record adverse events (AE): Possible AE will be evaluated and reported during the study, and their causal relationship with the components of the cream will be assessed. If an adverse reaction is found, the researcher will evaluate the intensity and frequency of each episode and decide whether or not additional treatments are needed. These side effects will be notified to the Andalusian Pharmacovigilance Center through the yellow card alert system. If the physician considers it appropriate, the patient will be withdrawn from the study.

#### Withdrawals or dropouts from the trial

Those patients meeting the selection criteria and willing to participate in the study who could not do so because they do not have the means to travel to the primary care center that corresponds to them will be offered the possibility of being followed up in the radiation oncology service. In addition, possible deviations from the protocol, discontinuities in visits, withdrawals, losses, and their reasons will be collected in a registry of incidents occurring during the follow-up of patients. In case the patient expresses her request to drop out of the trial for any reason, she will be withdrawn from the trial. Likewise, those patients who, by clinical criteria, the field investigator considers their discontinuation will be withdrawn. Patients lost to follow-up due to not attending the visits, death, and other reasons evaluated by the research team will also be recorded.

#### Outcomes

All outcomes are described in detail in Table 2. The primary outcome is the incidence of RD.

**Table 2:** Primary and secondary outcomes

	**Measuring instruments/definitions**
**Primary outcomes**	
Incidence of RD	Patients with radiation-induced dermatitis (RD)
Evaluation of the skin lesion (RD)	(1) remains the same or worsens; (2) partial improvement; (3) total improvement or complete cure
**Secondary outcomes**	
Onset and duration of RD	Time of onset and duration of post-radiation dermatitis
Toxicity in patients’ irradiated skin	Common toxicity criteria of the cooperative group for skin suggested by the RTOG Foundation (https://www.rtog.org) [18]
Discomfort and pain concerning symptoms and signs related to dermatitis	Degree of skin discomfort caused by radiation. Subjective assessment and quantification by the patient on an ordinal scale (0 to 10) Pruritus, pain, itching, or peeling of the skin
Quality of life	Quality of life of patients with skin conditions Skindex-29 questionnaire [19]

In addition, secondary outcomes include the level of toxicity in patients’ irradiated skin. It will be assessed according to the common toxicity criteria of the cooperative group for skin suggested by the RTOG Foundation (https://www.rtog.org) [18] according to which toxicity is classified into five levels: 0 for no toxicity or no change; 1 for scattered macular or papular rash or erythema that is asymptomatic; 2 for scattered macular or papular rash or erythema with pruritus or other associated symptoms; 3 for symptomatic generalized macular, papular, or vesicular rash; and 4 for exfoliative dermatitis or ulcerative dermatitis.

The assessment of discomfort and pain concerning symptoms and signs related to dermatitis is based on the subjective assessment and quantification by the patient on an ordinal scale (range 0 to 10), the Visual Analogic Scale (VAS), answering the following question: What score would you rate from 0 (where 0 is the situation where you do not feel symptoms such as pruritus, pain, itching, or peeling of the skin) to 10 (where 10 is the situation where you notice the most symptoms) to your skin problem where do you have the lesion?

Quality of life will be assessed by completing the Skindex-29 questionnaire. Skindex-29 is a self-administered questionnaire to measure the quality of life of patients with skin conditions every 4 weeks, consisting of 29 items. The Spanish version of Skindex-29 has been validated [19].

#### Sample size

Taking as reference those values provided in a previous study [20], the expected occurrence rate of RD (primary outcome variable) would be 46.7% in EG and 78.6% in CG, respectively; with a 1:1 allocation, accepting an alpha risk of 0.05 (5%) and a beta risk of 0.20 (20%), and assuming a follow-up loss rate of 20%, 88 eligible patients (44 in each group) are needed (calculations performed with the Granmo software version 7.12 in: https://www.imim.cat/ofertadeserveis/softwarepublic/granmo/, sample size for two independent proportions).

#### Recruitment

Patients from both groups will be recruited at the Radiation Oncology Unit of the Reina Sofia Hospital (Cordoba, Spain). They will be followed up by family physicians and a primary care occupational therapist after sessions 5, 10, and 15 of radiotherapy and 1 week after the end of the treatment. Those in the EG will be given the test cosmetic cream, while those in the CG will receive a moisturizing cream. The moisturizing cream will be very similar to that of the Alantel, both in color, container, weight, and smell, and will be manufactured by the laboratory producing Alantel.

## Assignment of interventions: allocation

### Sequence generation

The same number of participants will be assigned to the two study groups using the block randomization procedure. The assignation will be performed by one of the investigators, an expert in statistics, who will remain blind to the allocation of subjects during the statistical analysis. The random allocation sequence of eligible patients to the EG or the CG will be generated using EPIDAT version 4.1. Allocation will be implemented by submitting a written document with the randomized allocation sequence to be followed by each oncologist. Once the sequence for allocating the 88 patients to each group is generated prior to the start of the field phase, this will be distributed among the participating oncologists to prevent assigning the same code to two different patients. The numbers from 1 to 17 will be assigned to the oncologist one, from 18 to 32 will be assigned to the oncologist two, and so on. In this way, each oncologist will know which number and to which group each patient should be assigned, generating an alphanumeric code.

The alphanumeric codes will be constructed according to the following order: number of the recruited patient (01, 02,…); assigned group (A or B); oncologist responsible for the case (1–5); primary care physician (1–9).

## Assignment of interventions: blinding

This is a triple-blind study, where neither the investigator responsible for the intervention nor the patients included in the study will know at any time whether they receive Alantel cream or control moisturizing cream. The statistician in charge of the analysis will also remain blind. The product bottles will be similar in appearance, containing the active substances for the EG and a harmless moisturizing substance for the CG. The company making the Alantel cream will also prepare and provide the moisturizing cream.

## Data collection and management

The person in charge of monitoring the fieldwork of the study will contact the field researchers, if necessary, to complete the missing data in the data collection forms under the supervision of the principal investigator, who will not participate in the collection and mechanization of the data. After the trial, patients’ personally identifiable information will be removed and placed in a separate database for statistical analysis. The database that will be generated to process data from the case report form (CRF) will not collect any patient identification or demographic data. Each patient will be identified with an alphanumeric code, so the methodologist in charge of statistical analysis will be unaware of the patients’ identities. Only the members of the study management team (the principal investigator, the research oncologist in charge of the study in her Oncology department, the person in charge of the methodology), the research assistant in charge of data mechanization, and the person in charge of the Clinical Research and Ethics Committee who approved the trial will have access to the trial data set. The CRF will be safeguarded and kept in a place with appropriate security. Only individuals with permission granted by the principal investigator can access it.

The data collected in the CRF will be mechanized by a research assistant in a database created in Google Drive (Google Form tool). Only the principal investigator and the methodologist supervising the process and checking the data entered will have access, paying special attention to inconsistent data, outliers, and missing data.

The final data of the trial will be available to the project funder and the Ethics Committee. The personal information of the participants will be stored in a database that will be guarded by the principal investigator throughout the study period, safeguarding it for 5 years, as established by the Spanish legislation.

## Statistical methods

After the mechanization and filtering of the data, a descriptive analysis will be performed that will include the characteristics of the studied population (sociodemographic variables) and outcome variables using measures of central tendency, dispersion, and position in the quantitative variables and tabulation and calculation of relative frequencies in qualitative variables. The calculation of the main estimators with their corresponding confidence intervals for 95% safety will also be performed. An intention-to-treat analysis will be performed. To minimize the problem of missing data, the analysis will be performed by dragging the data from the visit from which they are obtained to the following visits and to the final one. That is, the intent-to-treat analysis will include all participants assessed at least once after randomization. In addition, a per-protocol analysis will be performed, defined as the group of participants who comply with the procedure to be followed, including the use of the substances tested in the study, in at least 80%. A pre-post-intervention comparative analysis will then be performed with the endpoints used; for this, we will use means comparison tests taking into account the results obtained in each of the visits (parametric, such as ANOVA for repeated measures, if the variables follow a normal distribution—Shapiro-Wilk Test—or non-parametric, such as Friedman’s test, if they do not follow it), or proportion comparison test on qualitative variables, such as Pearson’s chi-square test or Fisher’s exact test (*p* < 0.05). Finally, the results will be statistically compared between groups using the log-rank test after estimating the survival curve with the Kaplan-Meier method. Multiple linear regression analysis-dependent variable: results obtained with the VAS, and the survival analysis, by Cox regression-dependent variables: RD or no; RD improvement vs. no improvement and cured vs. non-cured will be performed to evaluate the effect of the product, adjusting for the sociodemographic and clinical variables presumably predictive or confounding. Statistical analysis will be performed using SPSS v.17.0 and EPIDAT 4.1 packages.

## Oversight and monitoring

### Composition of the coordinating center and trial steering committee

The Project Coordination Commission comprises the management team, which consists of 6 members who meet every 2 months to review the trial status. It is composed of the principal investigator of the project (Dr. Celia Jiménez, epidemiologist), Dr. Luis Angel Pérula (epidemiologist expert in methodology and statistics), Dr. María Espinosa (oncologist from the radiotherapy oncology service), and three family doctors (Dr. Gertrudis Montes, Dr. Juan José Muñoz, and Dr. Esperanza Romero).

Important modifications made to the initial protocol will be communicated to the funding body and the Ethics Committee and will be registered in clinicaltrials.gov.

### Adverse event reporting and harms

Possible AE will be evaluated and reported during the study, and their causal relationship with the components of the cream will be assessed. If an AE is found, the researcher will evaluate the intensity and frequency of each episode and decide whether additional treatments are needed. These AE will be notified to the Andalusian Pharmacovigilance Center and the Ethics Committee of the Reina Sofia Hospital (Cordoba, Spain). If the physician considers it appropriate, the patient will be withdrawn from the study.

## Dissemination plans

Study results will be communicated to clinical stakeholders and disseminated in communications at scientific events and through publications in peer-reviewed scientific journals. The final results will also be disseminated to the health professionals involved in the care of this type of patient through the internal communication channels of the Andalusian Health Service.

## Discussion

By carrying out this clinical trial, it is expected to verify that Alantel cream, based on natural products at high concentrations, has advantages over a moisturizing cream for the preventive and curative treatment of RD in patients with breast cancer. One of the main limitations of this study will be the time required to recruit the patients from the planned sample because the selection criteria are restrictive, and although the study is multicenter, recruitment will be coordinated through a single service of radiotherapy oncology. The COVID-19 pandemic caused the study to be delayed and could not start at the scheduled time. In addition, this influenced the patient recruitment process because accessibility to the health system was restricted and the research staff suffered an increase in the demand for care, focusing their efforts on the care of cancer patients with COVID-19.

## Trial status

The study was intended to start in 2019, but was interrupted due to the COVID-19 pandemic. The pilot study was conducted between July and September 2021, the training sessions for field researchers were held in October 2021, and the study concluded in June 2023.

## Abbreviations

RD: Radiation-induced dermatitis
EG: Experimental group
CG: Control group
GP: General practitioner
PC: Primary care
AE: Adverse events
